# Research Advances on Tilapia Streptococcosis

**DOI:** 10.3390/pathogens10050558

**Published:** 2021-05-06

**Authors:** Ze Zhang

**Affiliations:** 1 College of Life Sciences, Beijing Normal University, Beijing 100875, China; zhangze@nibs.ac.cn; 2 National Institute of Biological Sciences, Zhongguancun Life Science Park, Beijing 102206, China; 3 Tsinghua Institute of Multidisciplinary Biomedical Research, Tsinghua University, Beijing 102206, China

**Keywords:** tilapia, *Streptococcus agalactiae*, streptococcosis, virulence factors, subunit vaccine

## Abstract

*Streptococcus agalactiae*, often referred to as group B streptococci (GBS), is a severe pathogen that can infect humans as well as other animals, including tilapia, which is extremely popular in commercial aquaculture. This pathogen causes enormous pecuniary loss, and typical symptoms of streptococcosis—the disease caused by *S. agalactiae*—include abnormal behavior, exophthalmos, and meningitis, among others. Multiple studies have examined virulence factors associated with *S. agalactiae* infection, and vaccines were explored, including studies of subunit vaccines. Known virulence factors include capsular polysaccharide (CPS), hemolysin, Christie-Atkins-Munch-Peterson (CAMP) factor, hyaluronidase (HAase), superoxide dismutase (SOD), and serine-threonine protein kinase (STPK), and effective vaccine antigens reported to date include GapA, Sip, OCT, PGK, FbsA, and EF-Tu. In this review, I summarize findings from several studies about the etiology, pathology, virulence factors, and vaccine prospects for *S. agalactiae*. I end by considering which research areas are likely to yield success in the prevention and treatment of tilapia streptococcosis.

## 1. Introduction

*Streptococcus agalactiae* (*S. agalactiae*), also commonly referred to as group B streptococcus (GBS), is a severe pathogen that can infect humans and a diversity of other animals, including reptiles, frogs, bovines, fish (including tilapia), and pigs, among others [1,2,3]. Streptococcosis—the disorder caused by GBS infection*—*is a major obstacle faced by the tilapia aquaculture industry: in 2011, streptococcosis caused the loss of $40 million in the tilapia industry in China, owing to high morbidity and mortality, which can reach up to 80% in outbreaks [4,5]. Given this potential for loss, it is unsurprising that researchers are striving to develop effective means to control tilapia streptococcosis outbreaks. There has been some progress in research regarding serotypes and virulence mechanisms, and there are some promising prospects for effective vaccines; this review considers progress over the past several years from investigations about these topics.

GBS, like many other pathogenic species, possesses many virulence factors, biomolecular factors that promote the ability of pathogens to infect and/or damage hosts [6,7,8]. Among GBS virulence factors, a large number are known to affect adherence and invasion of host cells, as well as evasion of host immunity [7]. A better understanding of these virulence factors can support the development of control and therapeutic strategies. Studies of GBS isolated from humans have split virulence factors into pore-forming toxins, factors for immune evasion, resistance to antimicrobial peptides (AMPs), host-cell adherence and invasion, and other virulence factors [7,8,9]. Pore-forming toxins—vitally important pathogenesis factors—can facilitate the entry of GBS into host cells. The most common kinds of pore-forming toxins are β-hemolysin/cytolysin (β-H/C) and Christie-Atkins-Munch-Peterson (CAMP) factor. GBS can encode several virulence factors that promote immune evasion. The adherent factors, such as fibrinogen-binding protein (FbsA/B/C), laminin-binding protein (Lmb), the immunogenic bacterial adhesin (BibA), Pili (PilA/B/C), serine-rich repeat (srr), α C protein, and others, are responsible for GBS binding to extracellular matrix (ECM) components [7,8]. As a β-hemolytic bacterium, GBS has the hemolytic property, which is essential for immune evasion and further infection [7,8]. Immune evasion factors include C5α peptidase (ScpB), hemolytic pigment, superoxide dismutase (SodA), HylB, and so on. Several classes of resistance to AMPs factors have been discovered, like D-alanylation of LTA and penicillin-binding proteins (PBPs). In addition, pili possess the ability to mediate GBS resistance to AMPs [7,8,9]. Recently, other virulence factors, cyclic di-AMP and cell wall-anchored ectonucleotidase (CdnP) have been discovered [10].

The abuse of antibiotics to control streptococcosis can cause many problems, such as the resistance of strains [11,12]. Moreover, the application regimes for antibiotics can be inaccurate, and antibiotics can deleteriously affect food quality and safety. Vaccines—which induce adaptive immune responses—can overcome some of these challenges. Given their potent efficacy, the development of vaccines for preventing GBS outbreaks has been widely investigated, including inactivated vaccines, attenuated vaccines, subunit vaccines, and DNA vaccines.

## 2. The Etiology of Tilapia Streptococcosis

Several studies have sought to identify the pathogen responsible for tilapia streptococcosis. Although this topic has been controversial, it is now acknowledged that the major pathogen for tilapia streptococcosis affecting aquaculture is *Streptococcus agalactiae* (GBS) [13]. In addition, there is evidence that *Streptococcus iniae* can cause similar disease symptoms, and it is now clear that many aspects contribute to outbreaks of tilapia streptococcosis, including environmental conditions as well as the presence of certain viruses and fungi [14,15].

*S. agalactiae*, a Gram-positive bacterium, has a spherical shape with dimensions ranging from 0.2 to 1.0 microns in diameter [16]. When cultured, *S. agalactiae* may grow in pairs or chains. It is a facultative anaerobe and is catalase and oxidase negative; it possesses the capacity for lactic acid fermentation [17] and is classified into Lancefield group B streptococcus (based on the presence and type of its surface antigens) [18]. The serotypes of GBS strains are assessed based on a capsular polysaccharide antigen, and to date, GBS has been classified into ten kinds of distinct serotypes, including Ia, Ib, II-IX [19,20]. Among them, serotypes Ia, Ib, II, and III are the most prevalent in tilapia infections. Notably, in 2018, our group identified that among others also the IX serotype was an important agent in tilapia streptococcosis [11]. This serotype has the potential to become a major infectious strain for tilapia.

Tilapia streptococcosis is mainly observed in temperate and tropical tilapia-culturing areas, including China [13], Malaysia [21], Austria [22], Brazil [23], Columbia [24], and Thailand [25], among other areas. Typically, large-scale infections of tilapia will breakthrough in relatively warm seasons, especially summer. The mortality rate can reach 50–70% in less than a week [26].

Although there is variability among tilapia, some of the most common symptoms and pathological signs include erratic swimming and loss of orientation, unilateral or bilateral exophthalmia (also named “pop-eye”), anorexia, abdominal distention, darkening of the skin, and hemorrhaging skin around the anus or at the base of the fins, as well as abdominal dropsy, pale, but enlarged liver, inflammations around the heart and kidney, and meningitis [26,27]. There has been heated discussion about potential modes of transmission: the introduction of infected fish is the most common mode, yet there is also solid evidence supporting vertical transmission [26].

## 3. Virulence Factors

### 3.1. Capsular Polysaccharide

The capsular polysaccharide (CPS) is a pathogenic factor widely distributed among *Streptococcus* serotypes; this molecule comprises glucose, galactose, N-acetylglucosamine, and N-acetylneuraminate [28]. CPS has been a traditional epidemiological tool for investigating *S. agalactiae* infections in humans [29]; it is generally used for strain typing. CPS is known to contribute to disease severity [30], and molecular serotyping techniques have an elevated discriminatory power for epidemiological studies [31].

Experiments have shown that bacterial cells lacking the capacity to produce CPS lost their virulence in a neonatal rat model of lethal group B *Streptococcus* infection [32,33]. Similarly, using tilapia challenge assays, CPS-deficient GBS showed attenuated pathogenesis. And this mutant GBS was also cleared more easily from tilapia spleen tissue compared to the wild type GBS strain examined [34]. It is now understood that CPS can suppress the aggregation of the complement factor C3b to inhibit phagocytotic killing by host cells [35,36]. Sialylated CPS mimics cell surface carbohydrate epitopes and thereby decreases host immune recognition [37]. GBS is known to regulate CPS production in response to environmental signals to adjust the capacity for adherence and host invasion [38]. Further, Barato et al. (2016) used an infection model and showed that mutant GBS cells (unencapsulated) displayed increased adhesion to the tilapia intestinal epithelium [39].

### 3.2. CAMP Factor

The CAMP factor (also called co-hemolysin) is encoded by the *cfb* gene. This is an extracellular protein of 23.5 kDa [40] that functions to promote GBS pathogenesis [41,42,43]. In vivo assays have shown that partially purified CAMP factors can lead to death in rabbits [44]. Briefly, currently, understanding of the pathogenic impacts of CAMP includes its oligomerization to support forming discrete pores on host membranes and its binding to glycosylphosphatidylinositol (GPI)-anchored proteins, which can promote cell lysis [45]. Recently, two research groups identified the structure of CAMP, which provided more details about its perforating function [46,47]. Podbielski et al. (1994) demonstrated that a full-sized recombinant CAMP-factor exerts co-hemolytic activity [42]. Note that *cfb* is widely used as an identification marker for *Streptococcus agalactiae* due to its exclusive expression in GBS [40]; however, more recent studies have shown that there are homologs of *cfb* in *Streptococcus pyogenes*, *Listeria monocytogenes, Mobiluncus curtisis*, and *Propionibacterium acnes* [48,49].

### 3.3. HAase (Hyaluronidase)

Encoded by the *cyl*B gene, GBS promotes its invasion of hosts by secreting HAase to specifically hydrolyze the host-cell-wall component hyaluronic acid into unsaturated disaccharide units as the end product. Its degradative enzyme function by cleaving the glycosidic bond between N-acetyl-β-D-glucosamine and D-glucuronic acid residues destroys the host’s normal connective tissues and nervous system, which leads to expose to the host tissue cells to bacterial toxins and further facilitate deep tissue penetration during infection [50,51]. It is also now clear that GBS uses HAase to counteract host immune responses [52]. Whereas a host can normally promptly respond by generating hyaluronan (HA) polymers, from which small fragments ultimately combine with Toll-like receptors (TLRs) to elicit inflammatory responses, the secreted HAase from GBS degrades proinflammatory HA fragments down into their component disaccharides, thus blocking the host’s TLR2/4 signaling responses [52].

### 3.4. Cel-EIIB

The GBS phosphotransferase system (PTS) system, which is known to regulate bacterial virulence, can phosphorylated sugar substrates, including lactose, fructose, cellobiose, mannose, and sorbose [53]. Cellobiose-PTS (cel-PTS) is ubiquitously expressed in different serotypes of GBS, and strains genetically deficient for cel-PTS have decreased colonization ability and virulence [54]. One study showed that there are different expression levels of the cel-PTS component cel-EIIB between low and high virulence GBS [55,56]. Xu et al. (2018) reported that a cel-EIIB knockout strain showed a decreased ability of cellobiose utilization, as well as significantly reduced biofilm formation ability compared with the wild-type GBS strain [57]. It was also notable that knockout of cel-EIIB caused a 20% decrease in the accumulative mortality of tilapia due to GBS infection; the authors speculated that cel-EIIB knockout significantly decreased invasion and colonization efficiency [57].

### 3.5. LuxS/AI-2 Quorum Sensing System

Quorum sensing (QS) refers to a coordinated mode of gene expression regulation that supports bacterial communication and group activity [58]. The *LuxS* gene encodes S-ribosyl homocysteinase, which catalyzes the biosynthesis of the QS signaling molecule known as autoinducer 2 (AI-2), a furanosyl borate diester. *LuxS* is conserved among GBS serotypes and is ubiquitously expressed [59,60]. This 483 bp gene is composed of a conserved active center (H57, H61, C127) and a Zn^2+^-binding site (H-T-I-E-H) [61]. Ma et al. have reported that a mutant strain deficient for *LuxS* was defective for quorum sensing and displayed a more than 30-fold reduction in acid resistance compared to the wild-type strain [61]. The cell adherence was also decreased in the mutant strain. A study in tilapia showed a significantly decreased extent of infection and demonstrated that reintroducing *LuxS* into the *luxS* rescued hypervirulence [61].

### 3.6. Other Virulence Factors

Besides the aforementioned virulence factors, there are also other virulence factors, which have not been experimentally confirmed in tilapia GBS infections, but, which have been widely studied in mammalian GBS infections, including, for example, fibrinogen receptor (*FbsA, FbsB and FbsC)* [62]*,* superoxide dismutase (SOD), serine-threonine protein kinase (STPK), C5a peptidase *(scp)* [14]*, serine-rich repeat glycoproteins (srr1 and srr2)* [63]*,* β-hemolysin/cytolysin *(cylE)* [64], *pili* [65]*,* proteins Cα *(bca)* [66], *neul* [67], and α-like protein (*Alp*) [68], among others.

## 4. Progress Made in GBS Vaccinology

### 4.1. Inactivated Whole-Cell Vaccine

Formalin-inactivated and heat-killed whole-cell vaccines have been widely used in studies of pathogenic infection. Pasnik et al. (2005) conducted experiments wherein the relative percentage of survival (RPS) upon post-vaccination challenge was 49–50% [69,70]. Evans et al. (2004) performed similar assays using an inactivated whole-cell vaccination approach and challenge methods and recorded an RPS of 80% [71]. Thus, inactivated whole-cell approaches are useful for inducing immune responses to support basic studies and can confer protection against GBS infection (Table 1).

### 4.2. SAGs (S. Agalactiae Ghosts)

So-called *S. agalactiae* ghosts (SAGs) are empty cell envelopes from dead cells that have been explored as vaccine candidates owing to the presence of too many innate immunostimulatory agonists and their potent ability to activate innate and adaptive immunity responses. SAGs have been shown to induce cytokine secretion, which, in turn, contributes to the recruitment of T and B cells to lymph nodes. The recruitment of lymphocytes can increase the recognition of foreign antigens to elicit strong immune responses [72]. Wang et al. (2018) reported that immunization of tilapia with SAG elicited significantly higher resistance against GBS compared to the PBS-immunized controls [73] (Table 1). Moreover, SAG-immunized tilapia has stronger innate immunity (including phagocytic activity, lysozyme and superoxide dismutase activities) and adaptive immunity, especially IgM antibody titers. Finally, they found that the SAG-immunized tilapia showed significantly higher cytokine production (IL-1β, TNF-α, and TGF-β) than control animals.

### 4.3. Sip (Surface Immunogenic Protein)

There are some issues like inter-batch variability that can make use of inactivated whole-cell approaches less attractive than using vaccination approaches, which rely on more narrowly focused antigens (especially subunit vaccines) [74,75].

Sip is encoded by a gene in the GBS chromosome; this cell surface protein has been demonstrated as highly conserved; it is present in all GBS serotypes [76,77]. Given this broad distribution, it is unsurprising that Sip has been explored as a candidate for developing a subunit vaccine against GBS infection [78,79]. A 2014 study explored the immunogenicity of a Sip DNA vaccine, reporting an RPS value of 57% [79]; this was considered to represent relative effective immunoprotection. He et al. (2014) evaluated the truncated surface immunogenic protein (tSip) subunit vaccine and reported an RPS of 90% [78] (Table 1).

### 4.4. Surface Antigen Metabolic Enzymes

There have been attempts to assess the immunogenicity of enzymes present at the GBS surface, including phosphoglycerate kinase (PGK) [80], ornithine carbamoyl-transferase (OCT) [80], and the GapA subunit of glyceraldehyde-3-phosphate dehydrogenase of GBS [75,80]. PGK is responsible for its virulence and immunoprotective functions [81]. Using the recombinant PGK protein, Wang et al. (2014) found that PGK (as a subunit vaccine) against GBS infection of tilapia gave an RPS value of 82.4% [80]. The same study investigated OCT, which catalyzes the production of citrulline and phosphate from ornithine and carbamoyl phosphate substrates. Using recombinant OCT protein as a vaccine subunit gave an RPS value of 58.8%. Another study examining GapA as a subunit vaccine showed an RPS value of 63.3% [75] (Table 1).

### 4.5. Fibrinogen-Binding Protein A, α-Enolase, and GroEL

FbsA and enolase are adhesion proteins on the surface of bacteria. Yi et al. (2014) assessed potential immune protection against GBS infection upon vaccination with FbsA and Enolase, reporting RPS values of 40.63% and 62.50%, respectively [82]. The above data stated that the FbsA and enolase are multifaceted functions, including activating the host’s innate immune responses and relevant antibody responses and immunoprotection function. Thus, both of them can be efficient subunit vaccine candidates (Table 1).

GroEL is a heat shock protein (Hsp) of the chaperonin family of molecular chaperones; it is ubiquitously expressed in many bacteria [83]. This highly conserved chaperone functions in the proper folding of proteins. Li et al. (2019) reported that immunization of tilapia with GroEL (delivered with FC or FIC adjuvants) conferred protection against GBS, with an RPS value of 68.61% [74]. At the same time, which can increase antibody titers by promoting lymphocyte proliferation [74,84] (Table 1).

**Table 1:** Inactivated whole-cell vaccines and subunit vaccines explored for controlling tilapia streptococcosis.

Vaccine	RPS	Adjuvant	Vaccination	Challenge	Year	Reference
Inactivated	49–80%	Without	Intraperitoneal	Intraperitoneal	2004–2005	[69,70,71]
Inactivated	97%	Without/feed-based adjuvant	Oral	Intraperitoneal	2004	[71]
Sip	57%	--	Intragastrical	Intraperitoneal	2014	[79]
tSip	90%	FIA	Intraperitoneal	Intraperitoneal	2014	[78]
PGK	82.4%	Montanide ISA 763 AVG	Intraperitoneal	Intraperitoneal	2014	[80]
OCT	58.8%	Montanide ISA 763 AVG	Intraperitoneal	Intraperitoneal	2014	[80]
GapA	63.3%	FCA + FIA	Intraperitoneal	Intraperitoneal	2016	[75]
GapA	45.6%	Montanide ISA 763 AVG	Intraperitoneal	Intraperitoneal	2016	[75]
FbsA	40.63%	Adjuvant	Intraperitoneal	Intraperitoneal	2014	[82]
Enolase	62.50%	Adjuvant	Intraperitoneal	Intraperitoneal	2014	[82]
LIC	70%	Without	Feed-based	Intraperitoneally	2014	[85]
GroEL	68.61%	FCA + FIA	Subcutaneous	Intraperitoneally	2019	[74]
CWSAP 465	77.5%	FIA	Intraperitoneal	Intraperitoneal	2016	[86]
CWSAP1035	72.5%	FIA	Intraperitoneal	Intraperitoneal	2016	[86]
ISP	48.61%	FCA + FIA	Intraperitoneal	Intraperitoneal	2016	[87]
SAG	86.67%	--	Intraperitoneal	Intraperitoneal	2018	[73]

Notes: Inactivated—inactivated whole-cell vaccine, RPS—relative percentage of survival, FIA—Freund’s incomplete adjuvant, FCA—Freund’s complete adjuvant,—DNA vaccine without adjuvant, without—without adjuvant, LIC—cell wall surface anchor family protein, CWSAP—cell wall surface anchor family protein, ISP—immunogenic secreted protein.

### 4.6. Alternatives to Vaccines for Controlling Tilapia Streptococcosis

There has been research into other methods for managing, preventing, and treating tilapia streptococcosis, including intestinal microbiota modifier [88,89] and bacteriophages. Probiotics investigated to date include *Bacillus subtilis*, *Bacillus licheniformis*, and *Lactobacillus rhamnosus*. Liu et al. (2017) [90] reported that *Bacillus subtilis* could enhance tilapia growth, digestive enzyme activities, innate immune responses, and GBS resistance. Abarike et al. (2018) [91] reported that *Bacillus licheniformis* could also promote tilapia growth, immune responses, and GBS resistance. Xia et al. (2018) [92] showed supplementation with *Lactobacillus rhamnosus* increased tilapia growth, intestinal microbiota, immune responses, and GBS resistance. As for bacteriophages, Luo et al. (2018) [93] showed that tilapia treated with the bacteriophage HN48 had about 60% greater survival than control animals. Notably, there have been additional proposals about how to control GBS infections and tilapia streptococcosis, including, for example, the use of Chinese medicinal herbs [94] and alteration of water temperature [65,95], among others.

## 5. Conclusions and Prospects

This review has considered research progress into tilapia streptococcosis regarding etiology, pathology, epidemiology, and prospects for control, including immunology-based approaches. As genomics, transcriptomics, proteomics, metabonomics, and bioinformatics continue to mature, it is clear that ever-more-powerful tools can be applied to identify and better understand the biological basis and control prospects for GBS infections of many animals generally and of tilapia streptococcosis in particular [5,12,96]. At the same time, an increasing number of vaccine candidates and adjuvant (including Freund’s adjuvant, Montanide adjuvant, aluminum-based adjuvants and others) are being explored, and this is a promising area for control of disease outbreaks in aquaculture. There remain virulence factors of GBS isolated from tilapia, which are still not fully understood, so there is a knowledge gap for active research teams to fill. In the near future, it seems likely that antibiotic treatment will remain the main control approach; however, in the long run, as scientific research prompts breakthroughs in understanding and developing effective technologies, there may be some innovative treatments and control measures like vaccines plus probiotic and immune enhancers.
